# Case Report: Osteomyelitis of the Proximal Phalanx of the Finger in Patient With Ollier Disease

**DOI:** 10.3389/fsurg.2021.682101

**Published:** 2021-07-26

**Authors:** Konstantin Lipatov, George Melkonyan, Anatoly Shekhter, Artur Asatryan, Abduaziz Kholikov, Alexey Fayzullin

**Affiliations:** ^1^Institute of Clinical Medicine Named After N.V. Sklifosovsky, Sechenov First Moscow State Medical University, Sechenov University, Moscow, Russia; ^2^Institute for Regenerative Medicine, Sechenov First Moscow State Medical University, Sechenov University, Moscow, Russia; ^3^World-Class Research Center “Digital Biodesign and Personalized Healthcare”, Sechenov First Moscow State Medical University, Sechenov University, Moscow, Russia

**Keywords:** Ollier disease, surgical treament, hand surgery, enchonroma, osteomyelitis

## Abstract

Ollier disease is a rare congenital pathology characterized by the growth of enchondromas in bones, accompanied with their deformities, fractures, and the risk of malignancy. A 39-year-old patient with Ollier disease (acroform with lesions of hands and feet) suffered a rapid development of osteomyelitis of the proximal phalanx of the ring finger after a mosquito bite. The condition localized in the area of enchondroma. Surgical treatment included osteonecrectomy in the phalanx and enchondroma with excision of non-viable surrounding soft tissues, drainage of the surgical wound and the imposition of primary sutures. Morphological analysis confirmed the presence of ectopic embryonic cartilage specific for Ollier disease and the bone destruction. The excised tissues were infiltrated with immune cells and had signs of periosteal chronic inflammation including fibrosis and hyalinosis. These changes, which occurred long before the mosquito bite, became a favorable background for the development of a purulent infection.

## Introduction

Ollier disease, or dyschondroplasia, was described by a French surgeon Louis Ollier (1). It is a rare congenital disease with a prevalence of 1 per 100,000 (2). In this skeletal disorder, a part of the cartilage tissue retains its embryonic structure and does not transform into normal bone. As a result, focuses of cartilaginous tissue of various sizes accumulate among the bone tissue in proximity to a growth plate (3). Cartilage tissue often spreads beyond its normal localization. This migration can occur both inward and outward, accompanied by significant bone deformities. The pathologic cartilage affects bones by expanding their volume and making the cortical layer thinner (4). The tibia and femur, as well as the bones of the feet and hands are most commonly affected. This was reflected in Arenberg's classification (1964) distinguishing the four forms of Ollier disease: I – acroform with lesions of hands and feet; II – monomelic form affecting the bones of a single limb with the involvement of adjacent shoulder girdle or pelvis; III – unilateral or predominantly unilateral form; IV – bilateral form.

The Ollier disease is non-hereditary and associated with somatic gene mutations (IDH 1, IDH 2, PTHR 1) (5). In sporadic cases of Maffucci syndrome, enchondromatosis can develop with co-localized hemangiomas (6). Clinical manifestation of the Ollier disease include bone deformities associated with such complications as pathological fractures and malignant tumors, chondrosarcomas (7, 8).

To our knowledge, this is a first report of a purulent inflammation in the osteochondral structures affected by the Ollier disease. The following report demonstrates a case of osteomyelitis in a patient with Ollier disease, describes the surgical approach, and the outcomes.

## Case Report

A 39-year-old patient was admitted urgently to the purulent surgery department with complaints of pain in the base of the ring finger on the right hand and an increase in body temperature to 38°C. In anamnesis, the patient suffers from Ollier disease (acroform with lesions of the hands and feet) since childhood and had multiple surgeries to remove enchondromas on the feet. Seven days before the hospitalization, the patient was bitten by a mosquito at the area of enchondroma located on his right hand for many years. Inflammatory reaction manifested with pain syndrome, redness of the skin and signs of lymphangitis spreading to the middle third of the forearm. The outpatient surgeon diagnosed and opened the abscess bringing moderate relief to the patient on the 4th day of the disease. The patient was prescribed Amoksiklav (Amoxicillin/clavulanic acid), Nimesulide and daily dressings with Chlorhexidine.

**Figure 1:**
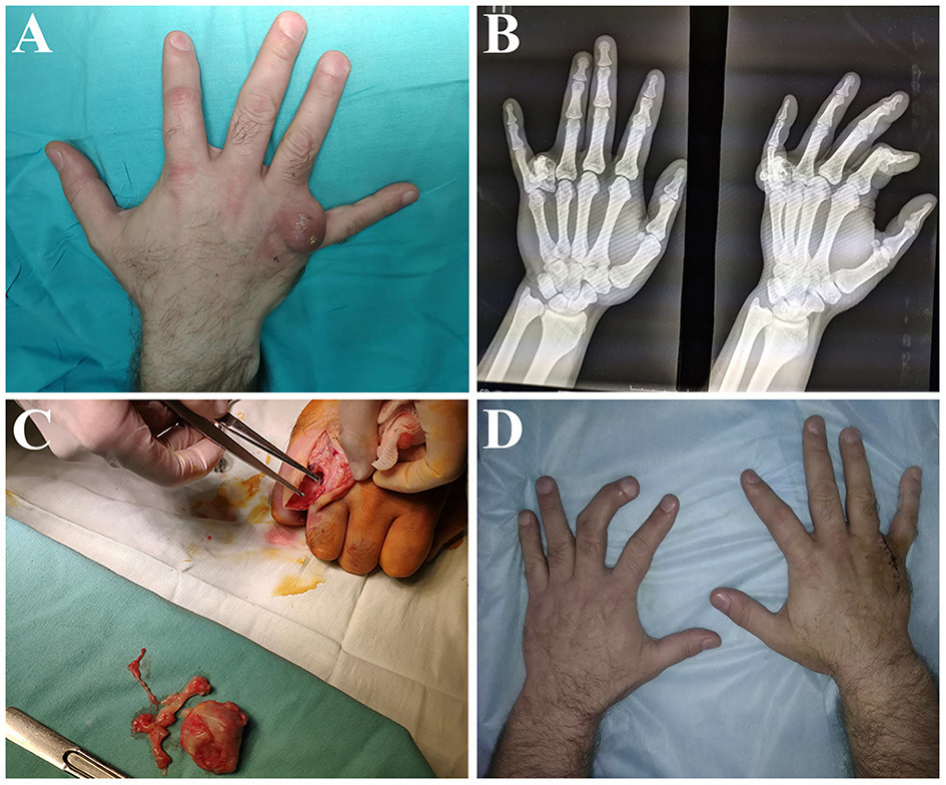
Diagnostics and surgical treatment. **(A)** Macroscopic observation of the hand on admission: a round infiltrated formation with a fistulous opening was located at the base of the ring finger. **(B)** X-ray of the hand on admission: an enchondroma with an heterogenous structure was visualized at the base of the proximal phalanx. **(C)** Intraoperative macroscopic revision of necrotic bone and soft tissues. **(D)** Macroscopic observation of the hand upon discharge from the hospital: wound healed by primary intention.

The improvement lasted for 3 days and was followed by an increase in pain and body temperature. On admission to the hospital, the deformity on the back surface of the right hand was painful, increased in volume (2.5 × 3.0 cm), with skin hyperemia and a fistula wound up to 0.5 cm containing dense serous-purulent masses (Figure 1A). Movements in the affected metacarpophalangeal joint were extremely limited. X-ray of the right hand revealed a round shape with bone density and heterogeneous structure on the proximal phalanx of the ring finger (Figure 1B).

Given the unfavorable course of the disease, the purulent focus was urgently treated under conduction anesthesia. During the intraoperative revision, it was revealed that the round-shaped bone-cartilaginous formation in the bottom of the operation wound was fused with the proximal phalanx. The central part of the formation consisted of gray necrotic tissue with purulent masses. The surrounding soft tissues were also in a state of chronic purulent inflammation. These findings let surgeons diagnose osteomyelitis of the proximal phalanx. The bone-cartilaginous formation was excised with the surrounding necrotic tissues (Figure 1C). The operation was completed with wound drainage and suturing. The postoperative period passed without complications. The wound healed by primary intention (Figure 1D). No signs of recurrence were evidenced in 9 months after the operation. The movement restrictions in the affected metacarpophalangeal joint remained. However, they were caused by Ollier disease long before the development of purulent inflammation. The patient was satisfied with the operation and did not report any adverse complications (Table 1).

**Table 1:** A table summarizing care checklist organized into a timeline.

**The event**	**Timeline**
The diagnosis of Ollier disease	20 years ago
Mosquito bite	Early 2020
Abscess treatment	7 days later
Recurrence of the symptoms and urgent operation	3 days later

**Figure 2:**
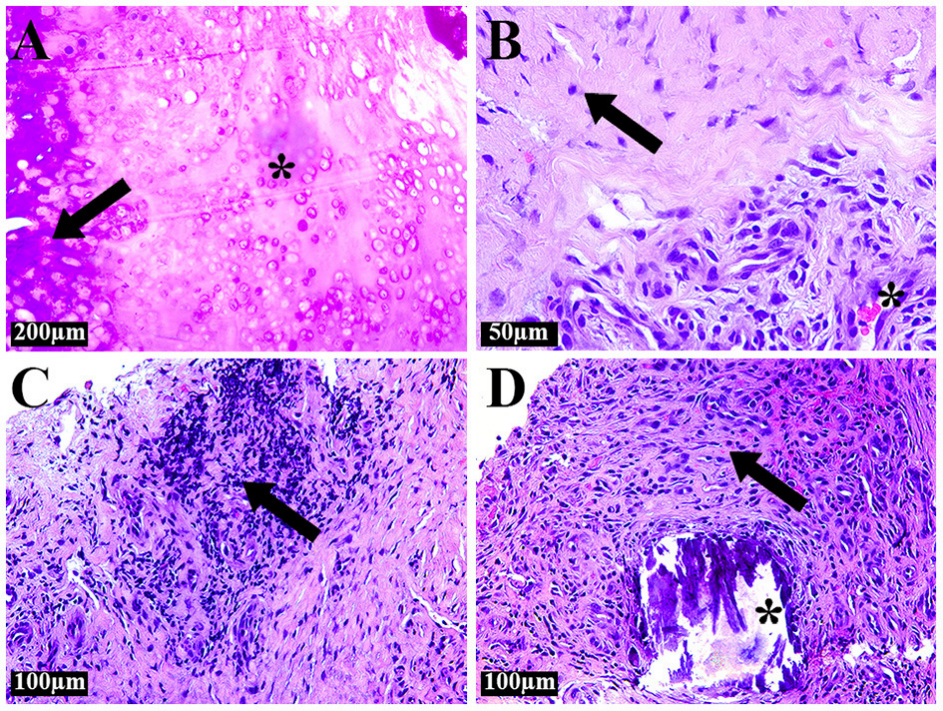
Histological examination of excised tissues. **(A)** Large area of embryonic cartilage (^*^). Small chondrocytes without lacunae, dystrophic changes in some cells, uneven distribution of chondrocytes, an increased amount of extracellular matrix. Small area of mature cartilage (arrow). **(B)** Fibrous connective tissue (arrow). Granulation tissue with numerous blood vessels (^*^). **(C)** Fibrous tissue infiltrated with immune cells (arrow). **(D)** Fragment of mineral deposition (^*^) surrounded by tissue infiltrated with leukocytes and macrophages (arrow).

A histological analysis of the excised tissues revealed that the bone tissue was in a state of necrosis. It was majorly replaced by an embryonic cartilaginous tissue (Figure 2A). The absence of nuclei in most chondrocytes indicated dystrophic changes in the cartilage. The centers of these areas were filled with mineral deposits. Extensive areas of embryonic cartilage tissue were closely connected to mature cartilage. This tissue differed from normal cartilage by its immature organization, uneven distribution and smaller size of chondrocytes, the absence of lacunae around them, and an increased content of the extracellular matrix. Examination of the excised periosteal soft tissues revealed foci of maturing granulation tissue with numerous vessels and scar-fibrous tissue with extensive areas of hyalinosis (Figures 2B,C). Tissue around the fragments of mineral deposits was infiltrated with leukocytes and macrophages (Figure 2D).

The histological examination of the excised tissues revealed the destruction and disorganization of the bone tissue with its partial replacement by embryonic cartilage and foci of mineral deposition. Purulent inflammation occurred not only in bone and cartilaginous structures but also in soft tissues.

## Discussion

Patients with Ollier disease have enchondromas of up to 50% of long and flat bones. No treatment is recommended if the lesions are radiographically benign and do not limit skeletal movements. The present report aims to include the risk of rapidly developing local infection to the fractures and malignization of which the patients are aware. Ollier disease can cause calcification, ossification and necrosis due to avascular cartilage promoting the development of local infection, which is what happened in the reported case. An exogenous infection (Staphylococcus aureus) entered the soft tissues with a mosquito bite resulting in an abscess of the soft tissues on the right hand. Despite the early surgical intervention, opening and drainage of the abscess, the infection rapidly transited to the osteomyelitis of the proximal phalanx of the finger affected by Ollier disease. Subsequent surgery revealed pronounced destructive changes in the phalanx, which occurred within only a few days from the moment of infection. The operation allowed the patient to return to his life with awareness of the risks associated with the condition.

In summary, the present report described a unique combination of the Ollier disease with osteomyelitis of the affected finger. The rare prevalence of the skeletal disorder led to an unexpected complication following the primary surgery. The chronic inflammation facilitated an extremely rapid spread of the minor purulent process to the bone and enchondroma. We recommend surgeons to take into account that even insignificant soft tissue infection can cause rapid development of osteomyelitis in patients with Ollier disease. Early antibacterial therapy and surgical resection of the affected tissue can prevent the condition.

## Data Availability Statement

The original contributions presented in the study are included in the article/supplementary material, further inquiries can be directed to the corresponding author/s.

## Ethics Statement

The studies involving human participants were reviewed and approved by Local Ethical Committee of Sechenov University. The patients/participants provided their written informed consent to participate in this study.

## Author Contributions

All authors listed have made a substantial, direct and intellectual contribution to the work, and approved it for publication.

## Conflict of Interest

The authors declare that the research was conducted in the absence of any commercial or financial relationships that could be construed as a potential conflict of interest.

## Publisher's Note

All claims expressed in this article are solely those of the authors and do not necessarily represent those of their affiliated organizations, or those of the publisher, the editors and the reviewers. Any product that may be evaluated in this article, or claim that may be made by its manufacturer, is not guaranteed or endorsed by the publisher.
